# Association between chronic idiopathic cough and sensitive skin syndromes is a new argument in favor of common neuropathic pathways: results from a survey on 4050 subjects

**DOI:** 10.1038/s41598-021-96608-w

**Published:** 2021-08-20

**Authors:** Laurent Misery, Jason Shourick, Grégory Reychler, Charles Taieb

**Affiliations:** 1 Laboratory of Neurosciences, University of Western Brittany, Brest, France; 2 Department of Dermatology, University Hospital of Brest, Brest, France; 3 EMMA, Fontenay-sous-Bois, France; 4 Department of Pneumology, University Clinics Saint-Luc, Brussels, Belgium

**Keywords:** Physiology, Diseases, Medical research

## Abstract

Sensitive skin syndrome has a neuropathic origin, which is why it is frequently associated with irritable bowel syndrome. We have looked for a possible association with chronic cough, which is commonly maintained by neurogenic mechanisms, whatever the initial cause(s). A survey was carried out on a representative sample of the population over 15 years of age using the quota method. The questionnaire included sociodemographic data and questions about sensitive skin, the presence of chronic cough, smoking and possible causes of chronic cough. Chronic cough was assessed by the Leicester Cough Questionnaire, and 4050 subjects responded (mean age: 45 years). Overall, 12.2% of subjects with a chronic cough were compared to the 87.8% without any cough. Among them, 72.5% had sensitive skin (vs. 47.8%, *p* < 0.001); additionally, 17.4% of the subjects with sensitive skin had a chronic cough (vs. 6.9% if no sensitive skin). These proportions were higher if very sensitive skin was reported. The risk of having chronic cough was twice as high if sensitive skin was reported [OR = 1.9 (1.5–2.4), *p* < 0.001]. The risk of having sensitive skin was also twice as high for chronic cough. Thus, chronic cough and sensitive skin are frequently associated. This association represents a new argument in favor of a neuropathic nature of sensitive skin. Sensitive skin and chronic cough are both modes of overreaction to environmental factors, which tend to be autonomized by neurogenic mechanisms. Dermatologists should ask their patients if they have a chronic cough, and pneumologists should ask about the presence of sensitive skin.

## Introduction

Cough is a defense reflex mechanism of the respiratory tract, with three phases: an inspiratory phase; a forced expiratory effort against a closed glottis; and opening of the glottis, with subsequent rapid expiration, which generates a characteristic cough sound^1^. Chronic cough is associated with many causes, such as chronic pulmonary diseases or psychogenic, mechanical or allergic disorders. Diseases causing chronic cough include asthma, eosinophilic bronchitis, gastro-esophageal reflux disease (GERD), postnasal drip syndrome or rhinosinusitis, chronic obstructive pulmonary disease (COPD), pulmonary fibrosis, and bronchiectasis. In a number of patients, no cause is identified, leading to the diagnosis of idiopathic cough^2^.

In these patients or in patients with chronic refractory cough, abnormalities of the neuronal pathways controlling cough are likely to be the primary disorder in these patients^3,4^. Agents classically used for treatment of neuropathic pain, such as gabapentin, pregabalin and amitriptyline, are frequently used to treat chronic cough. Following these successes, cough has emerged as a neuropathic disorder, and these drugs should be considered as antitussive treatments^5–7^. The diagnosis of cough hypersenitivity syndrome (CHS) was proposed for patients damage from chronic idiopathic cough, but also patients with other conditions who demonstrate a heightened response to inhaled cough-provoking stimuli such as citric acid or capsaicin^8^.

Because cough and itch share numerous mechanisms^9,10^, new arguments in favor of chronic cough as a neuropathic disorder should come from the skin.

Sensitive skin syndrome is a widely reported complaint but a diagnostic challenge because of its subjective symptoms and lack of clearly visible manifestations^11^. Sensitive skin syndrome can also be considered as a neuropathic disorder^12,13^, which is defined by the occurrence of unpleasant sensations (stinging, burning, pain, pruritus, and tingling sensations) in response to stimuli that normally should not provoke such sensations^14^. These unpleasant sensations cannot be explained by lesions attributable to any other skin disease. The skin can appear normal or can be accompanied by erythema. Sensitive skin can affect all body locations, especially the face.

Because sensitive skin is mainly characterized by a wide variety of sensory symptoms, it is highly likely that a neurosensory dysfunction in the skin represents one of the main pathological mechanisms of sensitive skin^15^. A systematic study that tested these pathophysiological hypotheses only found evidence for the neuronal hypothesis^16^, showing that the intra-epidermal nerve fiber density was significantly reduced, that means that nerve endings are damaged. A case–control quantitative sensory testing study demonstrated that the heat pain threshold was decreased in patients and that questionnaires assessed neuropathic pain^17^.

Hence, chronic cough and sensitive skin share many characteristics suggesting they are neuropathic disorders. They could be related to alterations of the peripheral nervous system, especially the nerve endings as well as central hypersensitivity^18^. Consequently, these syndromes could be associated in many cases. We have previously shown that neuropathic disorders such as irritable bowel syndrome^19^ or sensitive eyes^20^ were indeed frequently associated with sensitive skins. The aim of this study was to search for an association between chronic cough and sensitive skin by performing a large epidemiological survey and focusing on the characteristics of patients with both sensitive skin and chronic cough.

## Methods

As previously described^19^, a polling institute (HC Conseil Paris, France) performed the survey in May 2020**,** using a representative sample of the adult population (n = 4050) aged over 15 years old. Proportional quota sampling was applied to render the study population representative of the French general population aged > 15 years based on the National Institute for Statistics and Economic Studies Institute (INSEE) data. Email addresses of 900,000 internet users who agreed to participate in surveys (megabase-Quantar) were collected, with fixed quotas of subjects fulfilling predefined sociodemographical criteria applied according to sex, age and geographical areas. A double stratification according to age and gender, along with geographical residence area, was conducted. If the contact was unsuccessful or if he/she did not answer to all questions, another potential participant with the same characteristics was randomly selected. Each participant filled in a digital questionnaire containing approximately 30 items, taking approximately 12 min.

The first questions concerned socio-demographic characteristics and general health status aimed to avoid biased answers. Then, subjects were asked if they had cough for more than 3 weeks and if they categorized their skin as very sensitive, rather sensitive, not very sensitive or not at all sensitive. Putative causes of chronic cough were identified according to a list (see Table 1). Phototypes were defined according the usual classification^21^, from 0 (albinos) to 6 (black skin, black hairs, black eyes, no sunburn).

**Table 1:** Comparisons between ‘sensitive skin’ and ‘no sensitive skin’ groups.

	No sensitive skin	Sensitive skin	Effect size (mean difference (MD) or OR)	*p*
Age	48.6 years (+ 15.9)	42.2 years (± 15.6)	MD: 6.41 CI95%[5.44, 7.36]	< 0.001
Large towns	421 (21.1%)	616 (29.9%)	Reference	< 0.001
Urban areas	915 (46%)	797 (38.7%)	OR: 0.58 (0.49, 0.69)	
Rural areas	655 (32.9%)	646 (31.4%)	OR: 0.64 (0.53, 0.78)	
Phototype: I–II	617 (39.4%)	898 (58.4%)	Reference	< 0.001
Phototype: III–IV	855 (54.6%)	547 (35.6%)	OR: 0.44 (0.38, 0.51)	
Phototype: V–IV	94 (6%)	92 (6%)	OR: 0.67 (0.5, 0.91)	
Non smoker	1121 (56.3%)	1069 (51.9%)	Ref	< 0.001
Former smoker	507 (25.5%)	489 (23.7%)	OR: 1.09 (0.92, 1.29)	
Smoker	363 (18.2%)	501 (24.3%)	OR: 1.66 (1.38, 1.99)	
Chronic cough	136 (6.8%)	359 (17.4%)	OR: 3.44 (2.68, 4.43)	< 0.001
GERD	202 (10.1%)	313 (15.2%)	OR: 1.86 (1.16, 2.99)	< 0.001
Asthma	141 (7.1%)	364 (17.7%)	OR: 3.5 (2.1, 5.84)	< 0.001
COPD	59 (3%)	150 (7.3%)	OR: 3.16 (1.76, 5.66)	< 0.001
Other bronchic disease	29 (1.5%)	169 (8.2%)	OR: 6.09 (2.88, 12.91)	< 0.001
Chronic rhinitis	56 (2.8%)	224 (10.9%)	OR: 3.21 (1.87, 5.52)	< 0.001
ACEi use	473 (23.8%)	459 (22.3%)	OR: 1.54 (1.02, 2.32)	0.285
LCQ physical	4.8 (1.2)	3.8 (1.5)	MD: 1.02 CI95%[0.73, 1.3]	< 0.001
LCQ psychological	4.9 (1.4)	3.9 (1.5)	MD: 1.04 CI95%[0.78, 1.29]	< 0.001
lcq social	5.2 (1.4)	4 (1.7)	MD: 1.19 CI95%[0.89, 1.49]	< 0.001
LCQ total	14.9 (3.7)	11.7 (4.5)	MD: 3.25 CI95%[2.46, 4.04]	< 0.001

The validated French-speaking version of the Leicester Cough Questionnaire^22^ was used in patients reporing chronic cough. The Leicester Cough Questionnaire (LCQ) is a validated HRQoL questionnaire developed for patients presenting chronic cough^23^. The LCQ explores the impact of cough severity across physical, psychological and social domains and focuses specifically on cough in contrast to the other questionnaires that encompass multiple respiratory symptoms. It is a 19-item, self-completed questionnaire, divided into three domains. It contains a seven-point Likert-type response scale for each item. The LCQ score is calculated by aggregating the points assigned to each question in each domain and then dividing this total by the number of questions in the respective domain. The total severity score ranges from 3 to 21 and is calculated from 8, 7 and 4 items for physical, psychological and social domains, respectively. A lower score indicates a greater impairment of health status due to cough or sputum. It assesses the impact of symptoms over the last two weeks.

Quantitative variables were expressed as the mean and standard deviation. Categorical variables were expressed as frequencies and percentages. Comparisons between groups were performed using the Student T-test in the case of 2 groups of quantitative variables. For catagorical variables, intergroup comparisons were done with the χ2 test. Each variable was evaluated independently in a univariate analysis to identify factors associated with sensitive skin or chronic cough. Variables were retained in a stepwise manner for multivariate analysis. We conducted logistic regression using chronic cough and sensitive skin as outcomes. The level of significance was set at 0.05. Data were analyzed using R version 4.00.

### Patient and public involvement

The development of the research question and outcome measures were designed according to patients’ experience that they transmit to the authors of this study.

### Ethics declarations

In France, patient satisfaction surveys, experiments in human and social sciences in the field of health, evaluation of the practice of health professionals or teaching practices do not fall into the category of research covered by the Jardé law (not RPIH as defined by decree n° 2017-884 of 9 May 2017). Where the respondent is under 18 years of age, the parents or guardians must confirm their consent for the respondent to answer. The parent or legal guardian had the option of validating one of these three proposals: proposal 1: My child is between 15 and 18 years of age and I agree to them taking part in the survey; proposal 2: My child is unavailable, I will login later so they can take part in the survey; proposal 3; I don’t want my child to take part in the survey.

## Results

The sample was distributed with respect to quotas based on sex, age, socioprofessional status and regional distribution in the French sample population. Among the 4050 participants in the study, 2046 were women (50.5%) and 2004 were men (49.5%). The mean age was 45.4 years. Phototypes were distributed between I–II (1515 subjects, 48.8%), III–IV (1402 subjects, 45.2%) and V–VI (186 subjects, 6.0%).

In the studied sample, 864 (21.3%) were smokers, 996 (24.6%) former smokers and 2190 (54.1%) non-smokers. Notably, 515 participants (12.7%) reported GERD, while 505 (12.5%) reported asthma, 209 (5.2%) COPD and 198 (4.9%) other pulmonary diseases. Chronic rhinitis was reported by 280 subjects (6.9%) and the use of angiotensin-converting enzyme inhibitors (ACEi) by 932 (23.0%). Mean LCQ score was 12.6 ± 4.6 (social: 4.3 ± 1.7; psychological: 4.2 ± 1.6; physical: 4.0 ± 1.5).

**Figure 1:**
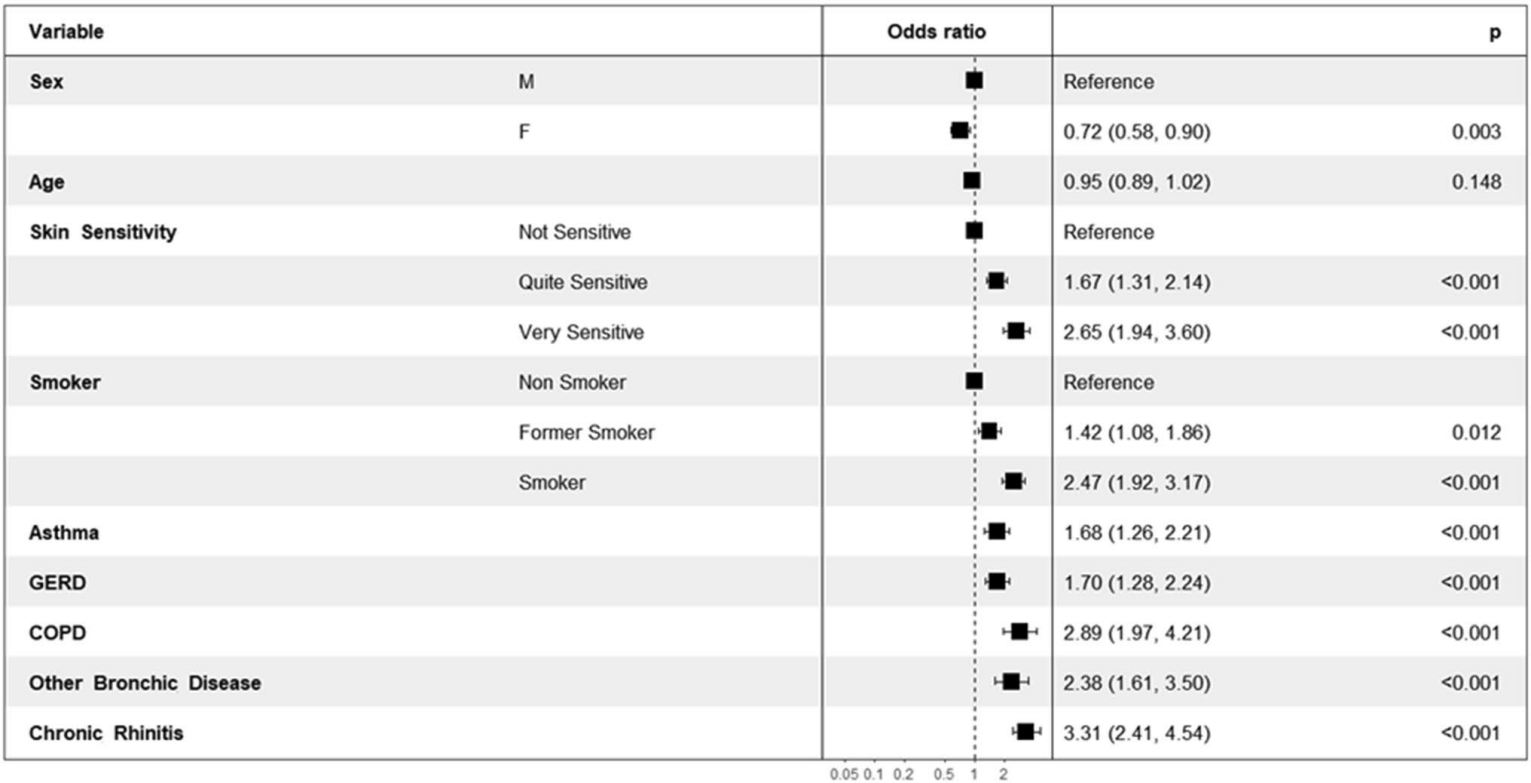
Multivariate analysis in patients with chronic cough.

A total of 495 participants reported chronic cough (12.2%), including 301 with dry cough (60.8%) and 194 with wet cough (39.2%). They were more frequently men (58.6%) than women (41.4%) (*p* < 0.001) and they were younger than subjects without chronic cough (42.2 vs. 45.8 years; *p* < 0.001). Patients with chronic cough more frequently lived in big towns (34.75 vs. 24.3%) than noncough subjects and less frequently in rural areas (36.2 vs. 43.1%) (*p* < 0.001). They were more frequently smokers (41.8 vs. 18.5%) or former smokers (25.45 vs. 24.5%) and less frequently non smokers (32.7 vs. 57.1%). They more frequently declared having GERD (29.3 vs. 10.4%), asthma (35. vs. 9.7%), COPD (23.4 vs. 2.6%), other pulmonary diseases (21.6 vs. 2.6%), chronic rhinitis (27.5 vs. 4.05%) and ACEi use (40.4 vs. 20.6%) (*p* < 0.001). They more frequently reported sensitive skin (72.5 vs. 47.8%, *p* < 0.001) (very sensitive skin: 28.7 vs. 10.5%, *p* < 0.001) and had lighter prototypes. Multivariate analysis confirmed the association of chronic cough and skin sensitivity with a gradient the more sensitive the skin is as OR for quite sensitive skin compared to not sensitive skin was 1.67 (1.31, 2.14) *p* < 0.001 and OR for quite sensitive skin compared to not sensitive skin was 2.65 (1.94, 3.6) *p* < 0.001 (Fig. 1).

**Figure 2:**
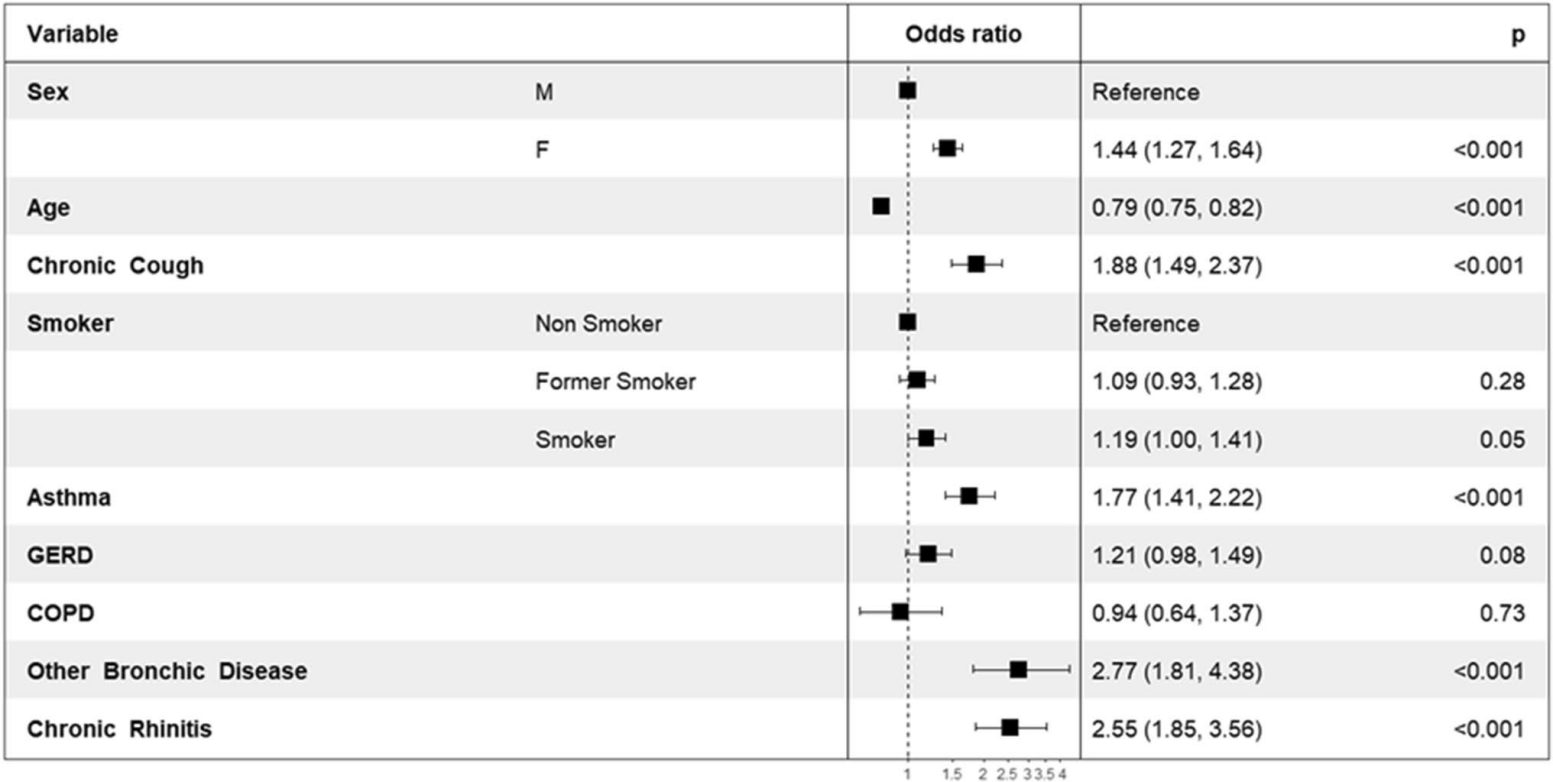
Multivariate analysis in subjects with sensitive skin.

A total of 2059 participants (50.8%) declared having sensitive skin (quite: 1544; very: 515) while 1991 did not declare having sensitive skin (49.2%). Detailed comparisons are given in Table 1. Subjects with sensitive skin were frequently women (54.0 vs. 47.0%, *p* < 0.001). They were younger, had lighter phototypes, and lived more frequently in large towns . They were more frequently smokers or former smokers . They more frequently reported chronic cough , as well as all associated causes, with the exception of ACEi use. There was no significant difference between wet and dry cough. LCQ gobal scores were not significantly different in terms of whether participants had sensitive skin but the physical, psychological and social subscores were lower in subjects with sensitive skin. Comparisons based on the severity of sensitive skin are given in Table 2 and showed gradients from non sensitive towards very sensitive skin. Multivariate analysis confirmed that chronic cough and sensitive skin were frequently associated: the subjects with sensitive skin reported chronic cough almost twice as much as subjects witout sensitive skin (OR = 1.88(1.49, 2.37)) (Fig. 2).

**Table 2:** Comparisons according to the severity of sensitive skin.

	No sensitive skin N (%) or M (SD)	Quite sensitive skin N (%) or M (SD)	Very sensitive skin N (%) or M (SD)	*p*
Males	1056 (53.0%)	735 (47.6%)	213 (41.4%)	< 0.001
Females	935 (47.0%)	809 (52.4%)	302 (58.6%)	
Age	48.6 (± 15.9) years	43.1 (± 15.9) years	39.7 (± 14.6) years	< 0.001
Large town	421 (21.15%)	421 (27.3%)	195 (37.9%)	< 0.001
Urban area	915 (46.0%)	633 (41.0%)	164 (31.8%)	
Rural area	655 (32.9%)	490 (31.7%)	156 (30.3%)	
Phototype: I–II	617 (39.4%)	637 (56.1%)	261 (64.9%)	< 0.001
Phototype: III–IV	855 (54.6%)	430 (37.9%)	117 (29.1%)	
Phototype: V–IV	94 (6.0%)	68 (6.0%)	24 (6.0%)	
Non smokers	1121 (56.3%)	829 (53.7%)	240 (46.6%)	< 0.001
Former smokers	507 (25.5%)	374 (24.2%)	115 (22.3%)	
Smokers	363 (18.2%)	341 (22.1%)	160 (31.1%)	
Chronic cough	136 (6.8%)	217 (14.05%)	142 (27.6%)	< 0.001
Dry cough	75 (55.15%)	126 (58.1%)	100 (70.4%)	0.017
Wet cough	61 (44.85%)	91 (41.9%)	42 (29.6%)	0.017
GERD	202 (10.15%)	204 (13.2%)	109 (21.2%)	< 0.001
Asthma	141 (7.1%)	233 (15.1%)	131 (25.4%)	< 0.001
COPD	59 (2.96%)	77 (4.99%)	73 (14.17%)	< 0.001
Other bronchial disease	29 (1.5%)	97 (6.3%)	72 (14.0%)	< 0.001
Chronic rhinitis	56 (2.8%)	129 (8.35%)	95 (18.45%)	< 0.001
ACEi	473 (23.8%)	321 (20.8%)	138 (26.8%)	0.011
LCQ physical	4.8 (± 1.2)	4.0 (± 1.4)	3.4 (± 1.6)	< 0.001
LCQ psychological	4.9 (± 1.4)	4.15 (± 1.45)	3.5 (± 1.6)	< 0.001
LCQ social	5.2 (± 1.45)	4.3 (± 1.6)	3.5 (± 1.7)	< 0.001
LCQ	14.9 (± 3.7)	12.47 (4.2)	10.5 (± 4.7)	< 0.001

## Discussion

To the best of our knowledge, this is the first study on the relationships between sensitive skin and chronic cough. The number of included subjects and the representativeness allowed us to assess that subjects with sensitive skin have a higher risk of damage from chronic cough and that patients with chronic cough more frequently have sensitive skin. Moreover, the risk for chronic cough was higher in subjects with very sensitive skin. Hence, sensitive skin should be considered as a skin equivalent of chronic cough, chronic rhinitis or asthma. Notably, chronic cough and sensitive skin are both more frequent in women than in men.

Smoking is a risk factor for sensitive skin^24^. A direct effect of tobacco smoke on the skin is probable. Nonetheless, an indirect effect through common pathogenic mechanisms of sensitive skin and chronic cough is also possible. These effects are probably not related to nicotine because nicotine is rather anti-inflammatory to the skin^25^.

ACEis are well known to be able to induce chronic cough^26^. This class of drugs blocks the conversion of angiotensin I to angiotensin II and prevents bradykinin breakdown. However, the lack of specificity of ACEis leads to the frequent side effects, such as cough and angio-edema. Other cutaneous reactions include pruritus, bullous eruptions, urticaria, other generalized rashes, photosensitivity and hair loss^27^. From our data, this chronic pruritus does not seem related to sensitive skin syndrome.

Recent advances in molecular neurophysiology provide knowledge to better understand the underlying mechanisms of unpleasant sensations. Hence, itch, pain and cough could share similar mechanisms^6,7,9,10^. Dysfunction of the nervous system, as manifested by neural plastic changes in primary sensory neurons of the peripheral nervous system (peripheral sensitization) and spinal cord and brain stem neurons in the central nervous system (central sensitization) will result in chronic cough, pain and itch. Inflammatory mediators can directly activate or sensitize sensory neurons in the peripheral and central nervous system, leading to cough, pain and itch hypersensitivity. Targeting these mediators or their receptors may allow control of inflammation, neuroinflammation and neuronal hyperexcitability. The efficacy of gabapentin and pregabaline on a number of patients suffering from chronic cough, itch and pain with (proved or not) neuropathic components is an example. Regarding the clinical aspects, similar mechanisms of peripheral and central sensitization may underlie symptoms such as hyperknesis and alloknesis, hyperalgesia and allodynia or hypertussivity and allotussivity^28,29^.

As one of the main causes of chronic cough^1,3,5^, neurogenic cough may be triggered by chemical (i.e., gastric acid reflux, perfumes, and other chemical odors such as cleaning agents), temperature (i.e., breathing cold or warm air), mechanical (i.e., talking, laughing, singing, touching neck, change in body position), or other (i.e., feeling of an abnormal sensation in throat—“tickle,” shortness of breath sensation) stimulation^5^. These triggering factors are obviously similar to those of sensitive skin^30^. Recent advances on chronic itch^31,32^ can be applied to the understanding of chronic cough and reciprocally.

The main limitation of the study is that data were only get thanks to the opinions of the participants because they were collected though an anonymous poll. This research process allows the obtention of numeours cases but further studies are needed on patients with confirmed diagnoses.

In conclusion, our study provides new arguments in favor of common mechanisms for sensitive skin and chronic cough since they are frequently associated and are two neuropathic disorders with a hyperreactivity to environmental factors. In both disorders, changes in the excitability and/or normal regulation of the peripheral and central neural circuits are instrumental in establishing hypersensitivity^33^. In clinical practice, dermatologists should ask their patients if they have a chronic cough, and pneumologists should ask about the presence of sensitive skin.

## Data Availability

We confirm the availability of the data on demand (charles.taieb@emma.clinic).
